# Accurate and equitable medical genomic analysis requires an understanding of demography and its influence on sample size and ratio

**DOI:** 10.1186/s13059-017-1172-8

**Published:** 2017-02-27

**Authors:** Michael D. Kessler, Timothy D. O’Connor

**Affiliations:** 1 Institute for Genome Sciences, University of Maryland School of Medicine, Baltimore, MD 21201 USA; 2 Department of Medicine, University of Maryland School of Medicine, Baltimore, MD 21201 USA; 3 Program in Personalized and Genomic Medicine, University of Maryland School of Medicine, Baltimore, MD 21201 USA; 4 University of Maryland Marlene and Stewart Greenebaum Comprehensive Cancer Center, Baltimore, MD 21201 USA

## Abstract

In a recent study, Petrovski and Goldstein reported that (non-Finnish) Europeans have significantly fewer nonsynonymous singletons in Online Mendelian Inheritance in Man (OMIM) disease genes compared with Africans, Latinos, South Asians, East Asians, and other unassigned non-Europeans. We use simulations of Exome Aggregation Consortium (ExAC) data to show that sample size and ratio interact to influence the number of these singletons identified in a cohort. These interactions are different across ancestries and can lead to the same number of identified singletons in both Europeans and non-Europeans without an equal number of samples. We conclude that there is a need to account for the ancestry-specific influence of demography on genomic architecture and rare variant analysis in order to address inequalities in medical genomic analysis.

The authors of the original article were invited to submit a response, but declined to do so. Please see related Open Letter: http://genomebiology.biomedcentral.com/articles/10.1186/s13059-016-1016-y

**Electronic supplementary material:**

The online version of this article (doi:10.1186/s13059-017-1172-8) contains supplementary material, which is available to authorized users.

Petrovski and Goldstein [1] recently reported on the analysis of a 5965-sample exome sequencing cohort that showed significantly different numbers of nonsynonymous singletons in Online Mendelian Inheritance in Man (OMIM) [2] genes across ancestry groups. More specifically, they showed significantly fewer singletons in Europeans, and they explained this as resulting from what they call a reduced access to ethnically matched controls. When they added 60,252 samples from the Exome Aggregation Consortium (ExAC) reference data set [3] to their analysis, the ancestry-based singleton distributions became more similar but were still significantly different across ancestries. The authors note that although this numerical difference across ancestries in singletons per individual may sound small, it can have a large impact on clinical interpretation and action.

While we concur with their overall conclusions, we would like to highlight that the ancestry-based differences that they observed are more complex and would not be addressed by equal representation across ancestries. Rather, it is important to consider recent demographic differences as they affect the distribution of rare alleles within a population. To demonstrate this, we ran simulations using ExAC allele frequencies of nonsynonymous OMIM [2] disease-gene variants to show that these demographic differences are a function of ancestry sample size and ratio (see Supplementary note in Additional file 1; scripts available upon request). Furthermore, our simulation results are consistent with recent findings about demographic history and allele frequency distribution [4–6]. We compared African, East Asian, South Asian, and Latino samples with (non-Finnish) European samples, and then down-sampled from the ExAC reference cohort to show what candidate variant analysis would look like in studies with diverse cohorts of varying sample sizes. Since our results are qualitatively the same for each of the ancestries when compared with Europeans, we describe the results from our analysis of African and European samples as a representation of the population-based pattern, and present other comparisons in Additional file 1: Figures S1–S6.

When African and European sample totals are equal, the difference in singletons per individual persists at low sample sizes and is reduced to zero as the number of African and European samples in the analysis cohort each reaches 1000 (Figs. 1 and 2). As these African and European sample totals each reach 5200 (close to the maximum number of African samples in the ExAC reference data set), the number of singletons in Africans becomes significantly lower than the number in Europeans (Figs. 1 and 2). This is consistent with observations from recent large sequencing studies that show that ultra-low frequency variants are more prevalent in individuals of predominantly European ancestry than in individuals of predominantly African ancestry as a result of differences in population growth in the past 10,000 years [4–7].

**Fig. 1:**
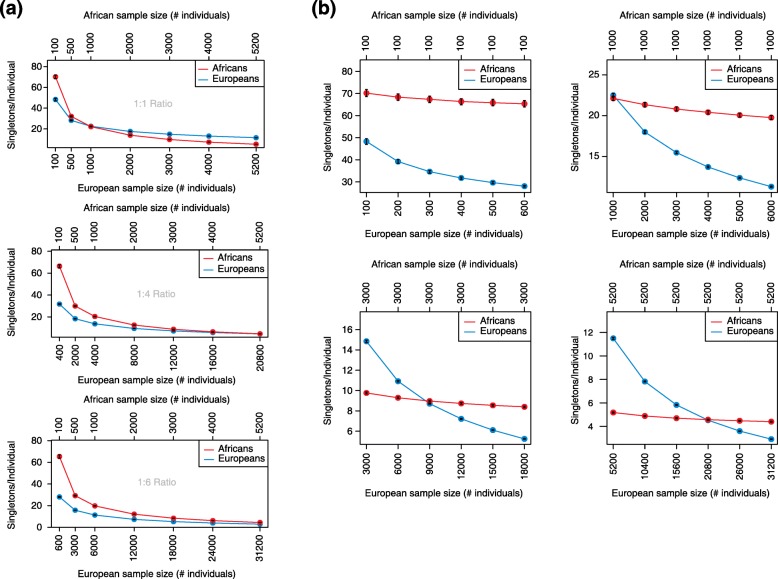
Simulated numbers of singletons per individual across sample size and ratio in Africans and Europeans. **a** The number of simulated singletons per individual is shown for Africans (*red*) and Europeans (*blue*) at different population sizes. Each panel has a different constant ratio of African to European sample size. *Black error bars* represent a 95% confidence interval from 200 replicates. **b** The number of simulated singletons per individual is shown for Africans (*red*) and Europeans (*blue*). However, African sample size is held constant in each panel, with African to European sample size ratio varying along the x-axis. *Black error bars* represent a 95% confidence interval from 200 replicates

**Fig. 2:**
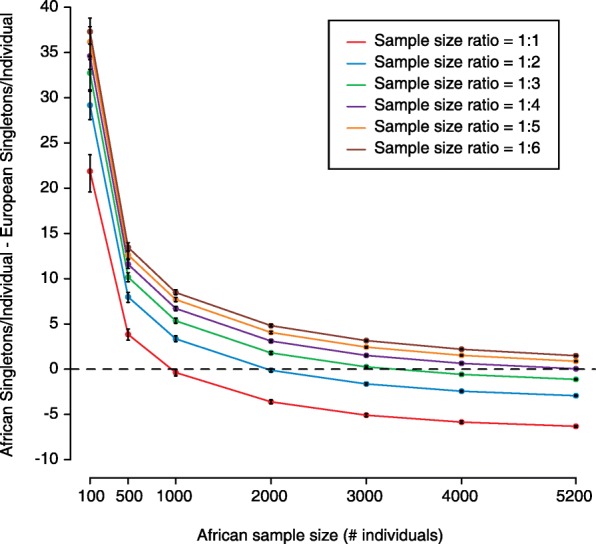
Difference between Africans and Europeans in number of singletons per individual across sample size and ratio. The difference between African-simulated singletons per individual and European-simulated singletons per individual is plotted along the y-axis. African sample size varies along the x-axis and each *colored line* represents a different ratio of African to European sample size. *Black error bars* represent a 95% confidence interval from 200 replicates

Another key variable is the ratio between African and European sample sizes. As the ratio of African to European sample number decreases, the number of singletons per individual decreases by more in Europeans than in Africans (Fig. 1b). Therefore, when this ratio is low, as is usually the case because of the Eurocentricity of most major sequencing studies, Europeans have a comparatively reduced number of singletons. Our simulation results suggest that researchers usually observe this reduced number of singletons in Europeans compared with Africans as a result of both low sample size ratio and moderate overall sample size (this holds for other less-represented populations as well). Clinically, this becomes a challenging discrepancy that leads to the costly need to adjudicate additional candidate variants in individuals of non-European ancestry [1, 8]. However, our simulations demonstrate that despite a low ratio of African to European sample size, the difference between populations in singletons per individual goes away as the African population size becomes large enough (Figs. 1 and 2).

This impact of sample size and ratio can help to explain the difference across ancestry in singletons per individual seen by Petrovski and Goldstein [1]. In their initial analysis, the ratio of African to European sample size was 1:10.05 and the sample sizes themselves were relatively small (505 Africans and 5094 Europeans). When they included the ExAC reference data set in their analysis, the population size ratio increased to 1:6.74 and the overall sizes also grew (to 5708 Africans and 38,464 Europeans). Our simulations show that both of these changes will reduce the number of singletons in African individuals by more than that in European individuals, as is seen in the results published by Petrovski and Goldstein [1]. However, had the ExAC data included in the analysis resulted in the appropriate sample size and/or ratio, the pattern they highlight would have disappeared or even reversed. While we strongly agree with the need to increase the representation of non-Europeans in sequencing studies and the need to further understand the impact of ancestry-specific genomic patterns [8], our results support the need to consider population-specific allele distributions (i.e., site frequency spectra) when establishing population proportions within a study. By doing this, differences between Europeans and underrepresented populations can potentially be addressed without the inclusion of equal numbers of samples from each population. Overall, we applaud the efforts of Petrovski and Goldstein to highlight the need to make resources equally useful to all. In order to take a significant step forward in reaching this goal, we must account for the impact of demographic history and how it shapes inter-population differences in allele frequency distributions.

## Additional file

Additional file 1: Supplementary note and figures. The supplementary note describes the simulations we performed and the figures are analogous to the main text figures but represent data from simulations done with Latino, South Asian, and East Asian allele frequencies. (PDF 666 kb)

## Abbreviations

ExAC: Exome Aggregation Consortium
OMIM: Online Mendelian Inheritance in Man
