# Supplementary material for: Accurate and equitable medical genomic analysis requires an understanding of demography and its influence on sample size and ratio
Source: Genome Biol. 2017 Feb 27;18:42. doi: 10.1186/s13059-017-1172-8 (PMC5330117; doi:10.1186/s13059-017-1172-8)

Supplementary note: simulations using ExAC allele frequencies

A VCF file containing variant sites from the Exome Aggregation Consortium (ExAC) data set was downloaded from the Broad Institute's ExAC web browser [1]. Per population allele frequencies from these VCF files were then used to simulate variant counts per population according to a binomial distribution. Each analysis consisted of 200 replicates of simulated counts per nonsynonymous Online Mendelian Inheritance in Man (OMIM) disease-gene variant for non-Finnish Europeans (NFE in ExAC) and a single non-European population (African, Latino, South Asian, or East Asian). Replicates in which one population had a variant count of one and the other population had a count of zero contributed to the singleton counts. A variant was considered an OMIM disease-gene variant if it was within a gene that is associated with disease according to the OMIM database [2] annotations.

References

1. Lek M, Karczewski KJ, Minikel EV, Samocha KE, Banks E, Fennell T *et al.* Analysis of protein-coding genetic variation in 60,706 humans. Nature. 2016;536:285–91.
2. Amberger JS, Bocchini CA, Schiettecatte F, Scott AF, Hamosh, A. OMIM.org: Online Mendelian Inheritance in Man (OMIM(R)), an online catalog of human genes and genetic disorders. Nucleic Acids Res. 2015;43:D789–98.

Supplementary figures

Fig. S1. a The number of simulated singletons per individual is shown for Latinos (red) and Europeans (blue) at different population sizes. Each panel has a different constant ratio of Latino to European sample sizes. Black error bars represent a 95% confidence interval from 200 replicates. **b** The number of simulated singletons per individual is shown for Latinos (red) and Europeans (blue). In these graphs, Latino sample size is the same within each panel, whereas European sample size (and thus the ratio of the two sample sizes) varies along the x-axes. Black error bars represent 95% confidence intervals from 200 replicates.

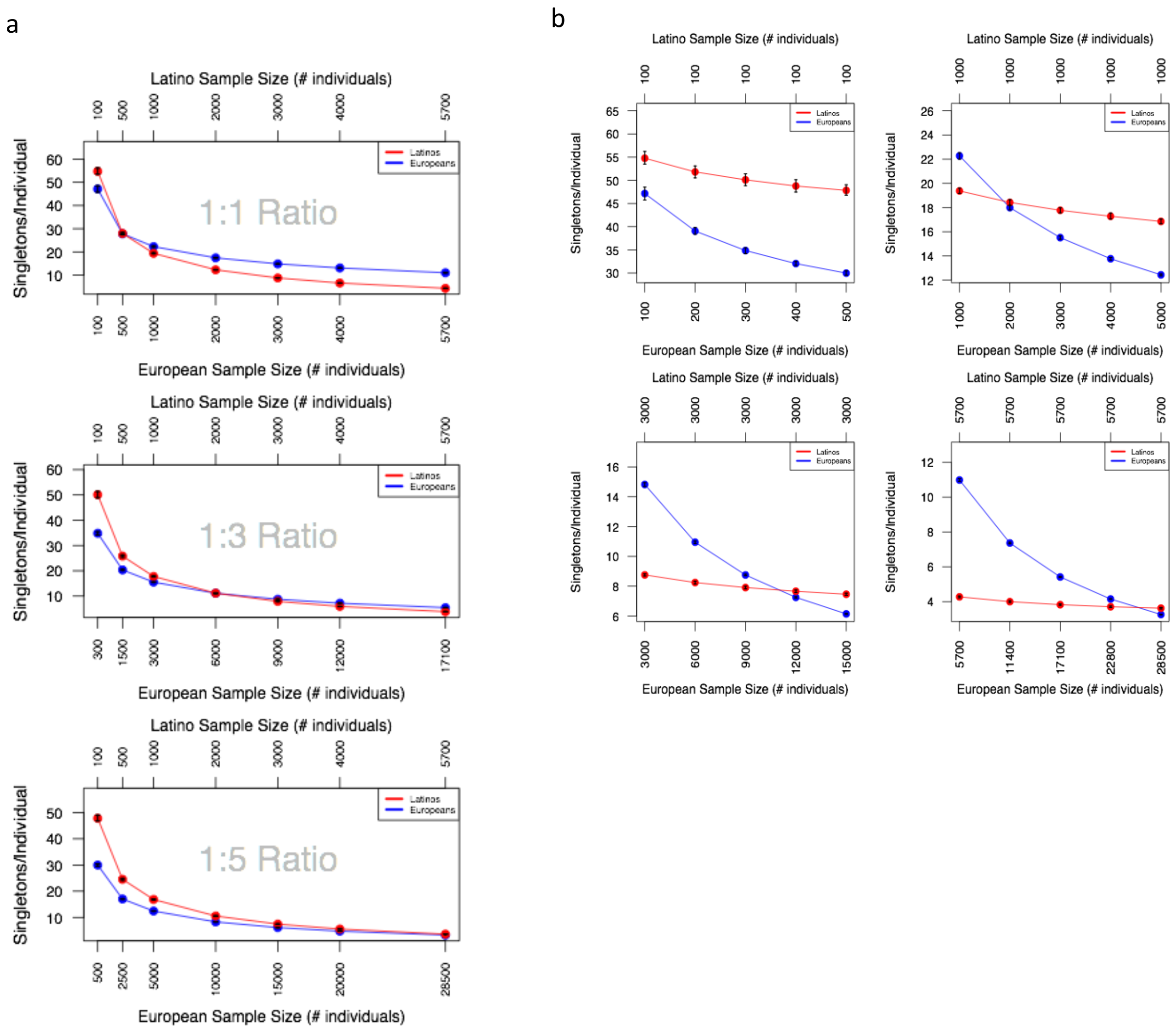

Fig. S2. The difference between Latino-simulated singletons per individual and European-simulated singletons per individual is plotted along the y-axis. Latino sample size varies along the x-axis and each colored line represents a different ratio of Latino to European sample sizes. Black error bars represent a 95% confidence interval from 200 replicates.

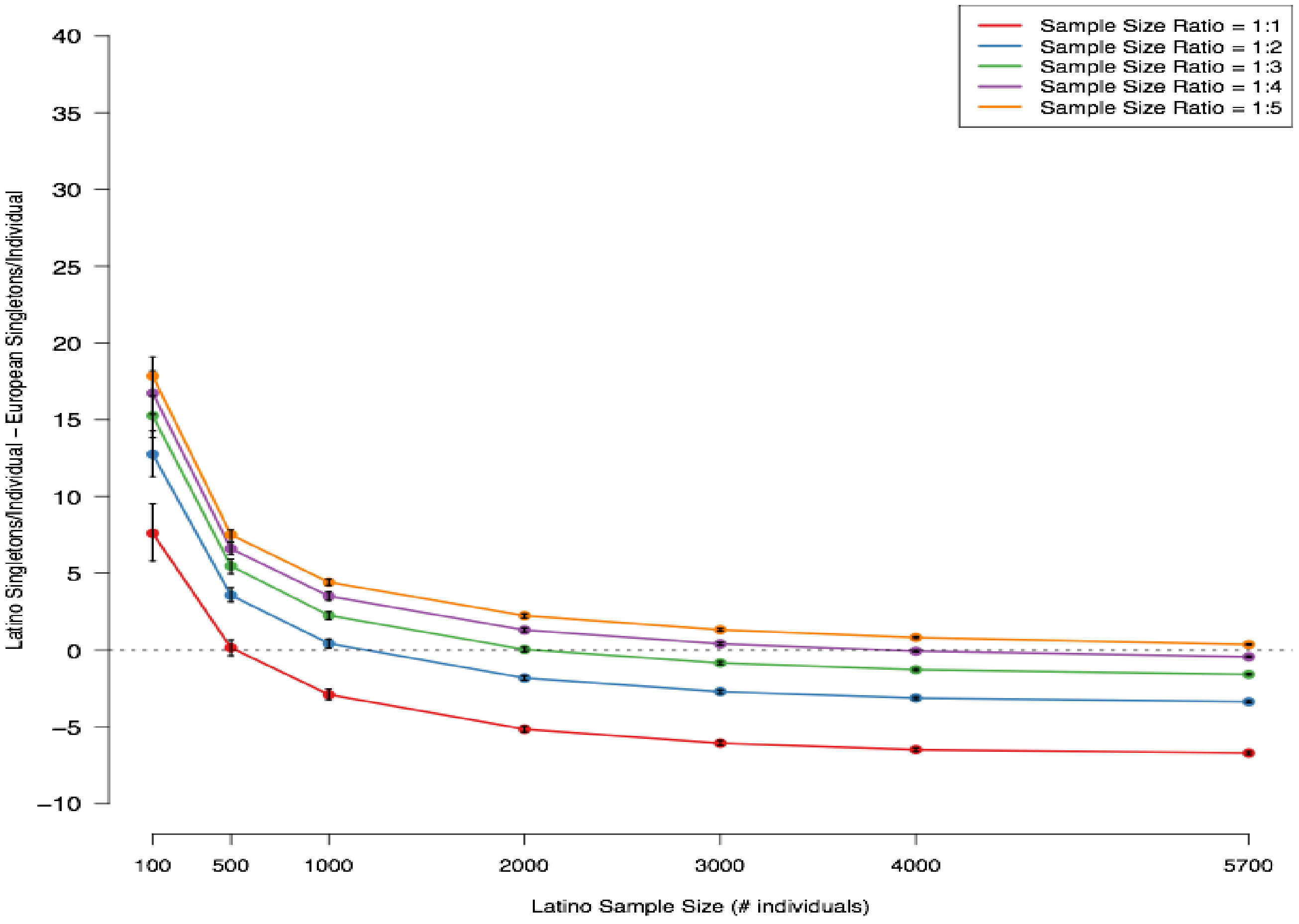

Fig. S3. **a** The number of simulated singletons per individual is shown for South Asians (red) and Europeans (blue) at different populations sizes. Each panel has a different constant ratio of South Asian to European sample sizes. Black error bars represent 95% confidence intervals from 200 replicates. **b** In these graphs, the number of simulated singletons per individual is shown for South Asians (red) and Europeans (blue). However, South Asian sample size is held constant in each panel, with European sample size (and thus the ratio of the two sample sizes) varying along the x-axes. Black error bars represent 95% confidence intervals from 200 replicates.

a b

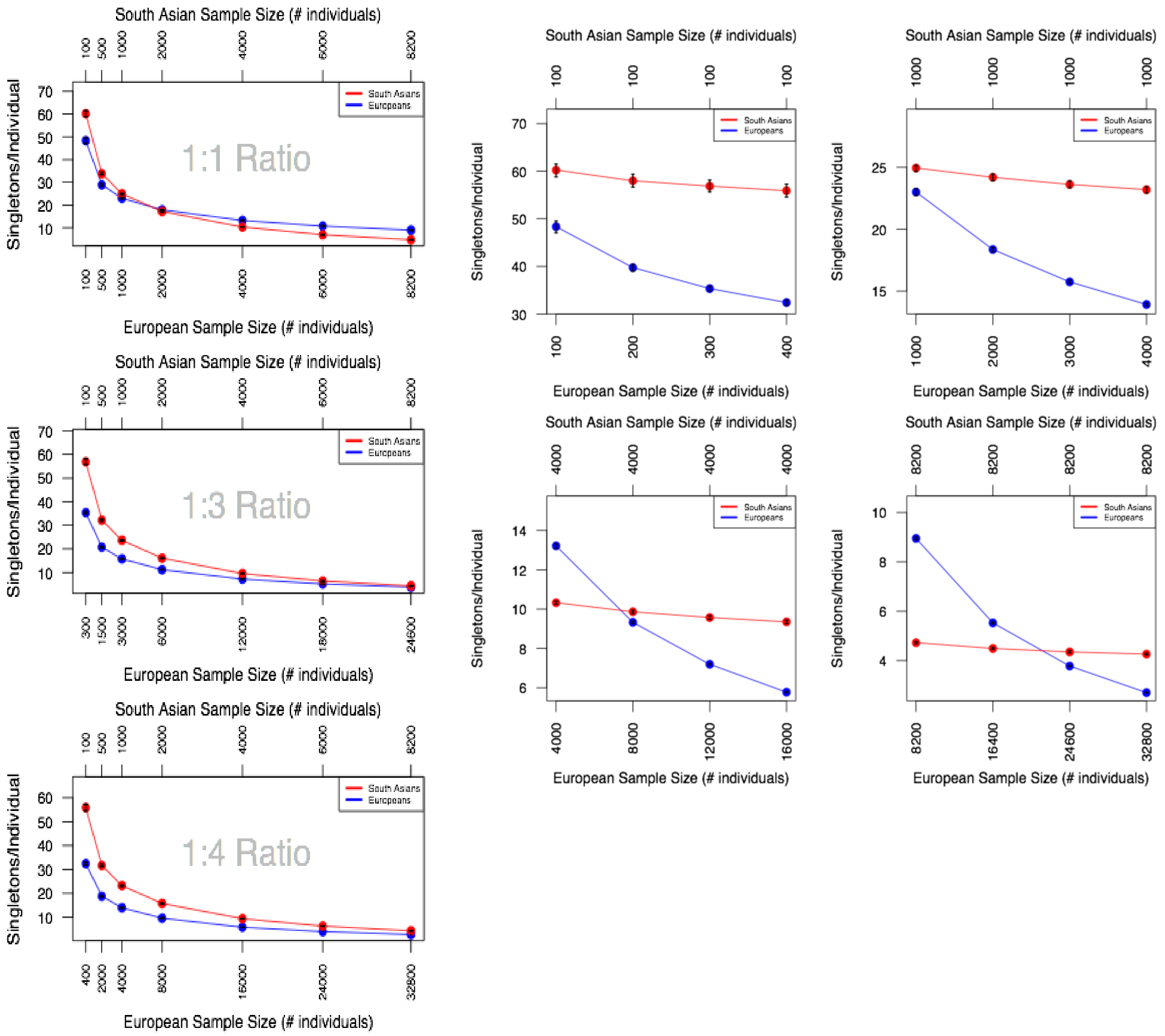

Fig. S4. The difference between South-Asian-simulated singletons per individual and European-simulated singletons per individual is plotted along the y-axis. South Asian sample size varies along the x-axis and each colored line represents a different ratio of South Asian to European sample size. Black error bars represent 95% confidence intervals from 200 replicates.

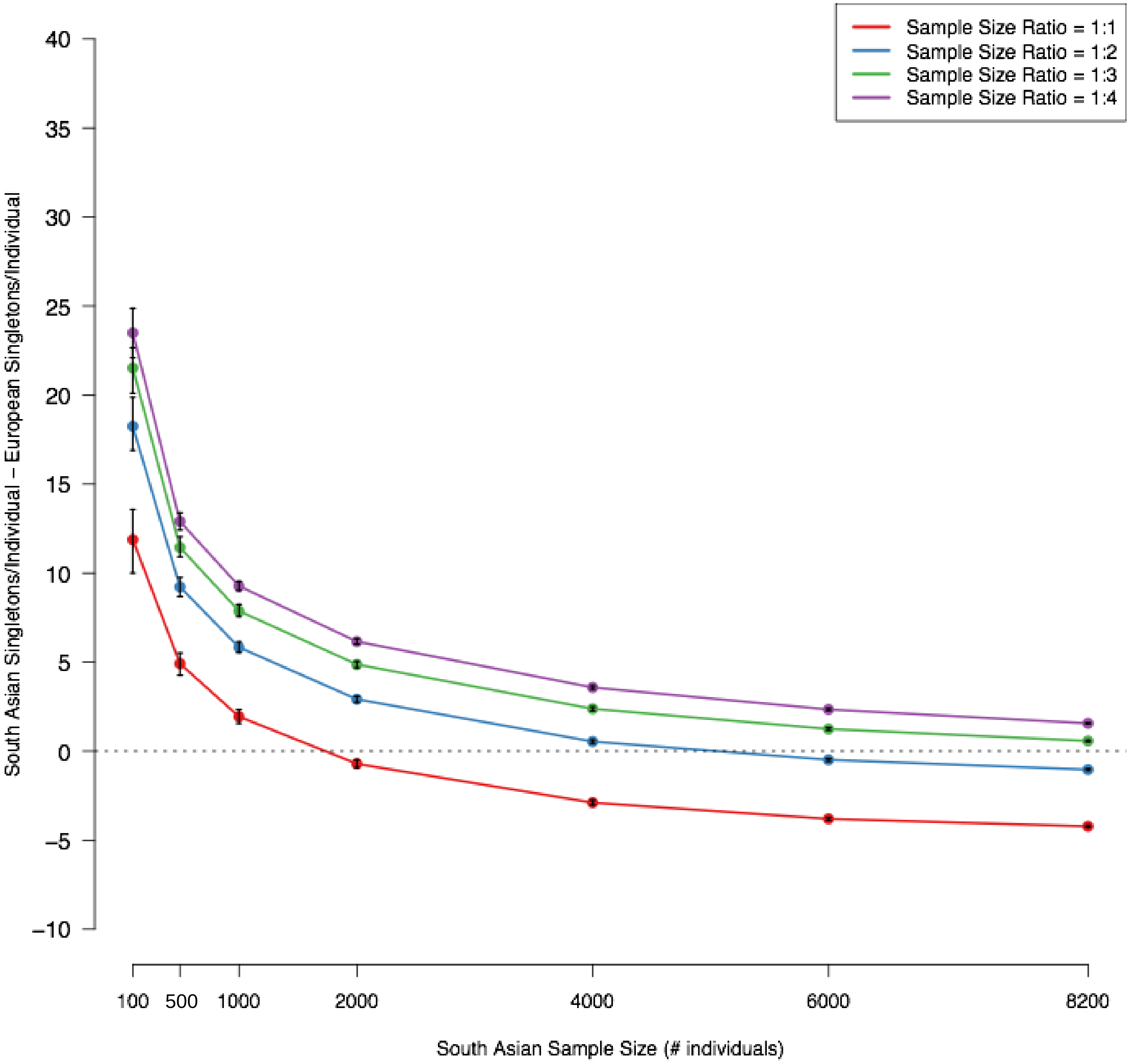

Fig. S5. **a** The number of simulated singletons per individual is shown for East Asians (red) and Europeans (blue) at different populations sizes. Each panel has a different constant ratio of East Asian to European sample sizes. Black error bars represent 95% confidence intervals from 200 replicates. **b** In these graphs, the number of simulated singletons per individual is shown for East Asians (red) and Europeans (blue). However, East Asian sample size is held constant in each panel, with European sample size (and thus the ratio of the two sample sizes) varying along the x-axes. Black error bars represent 95% confidence intervals from 200 replicates.

a

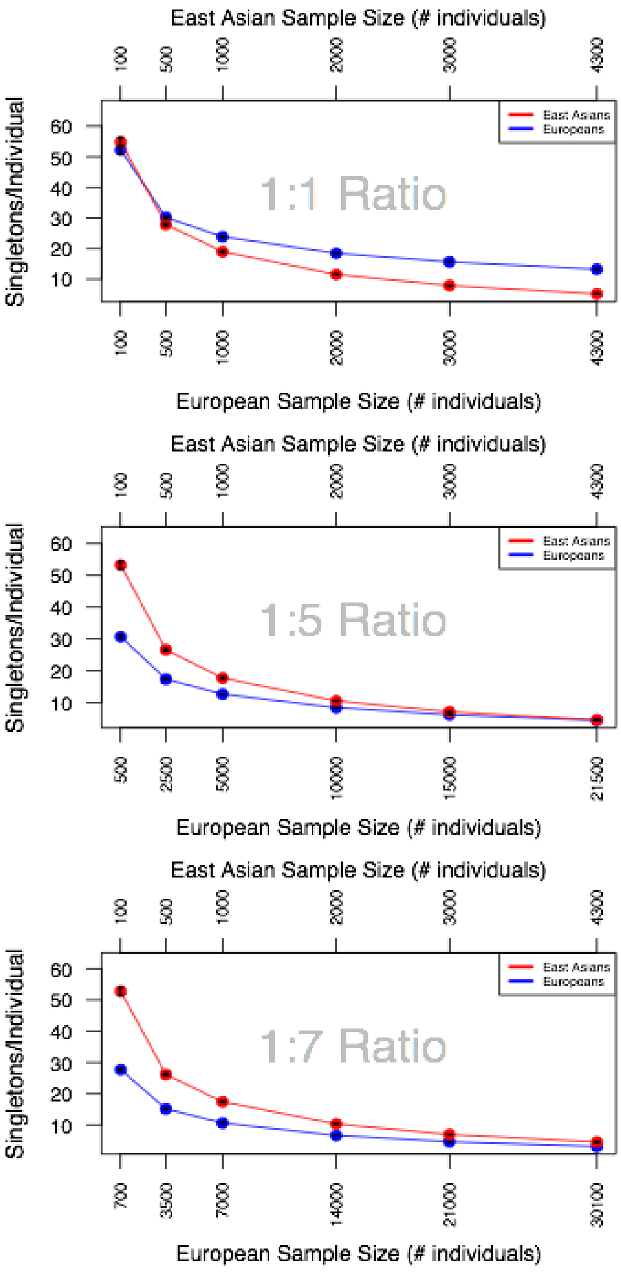

b

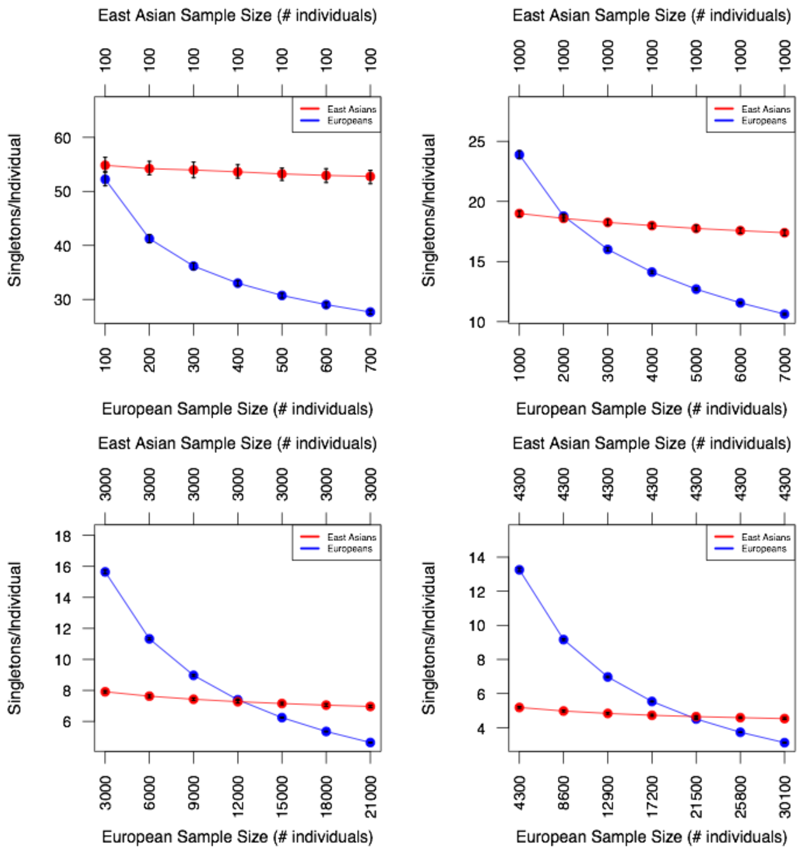

Figure S6. The difference between East-Asian-simulated singletons per individual and European-simulated singletons per individual is plotted along the y-axis. East Asian sample size varies along the x-axis and each colored line represents a different ratio of East Asian to European sample sizes. Black error bars represent 95% confidence intervals from 200 replicates.

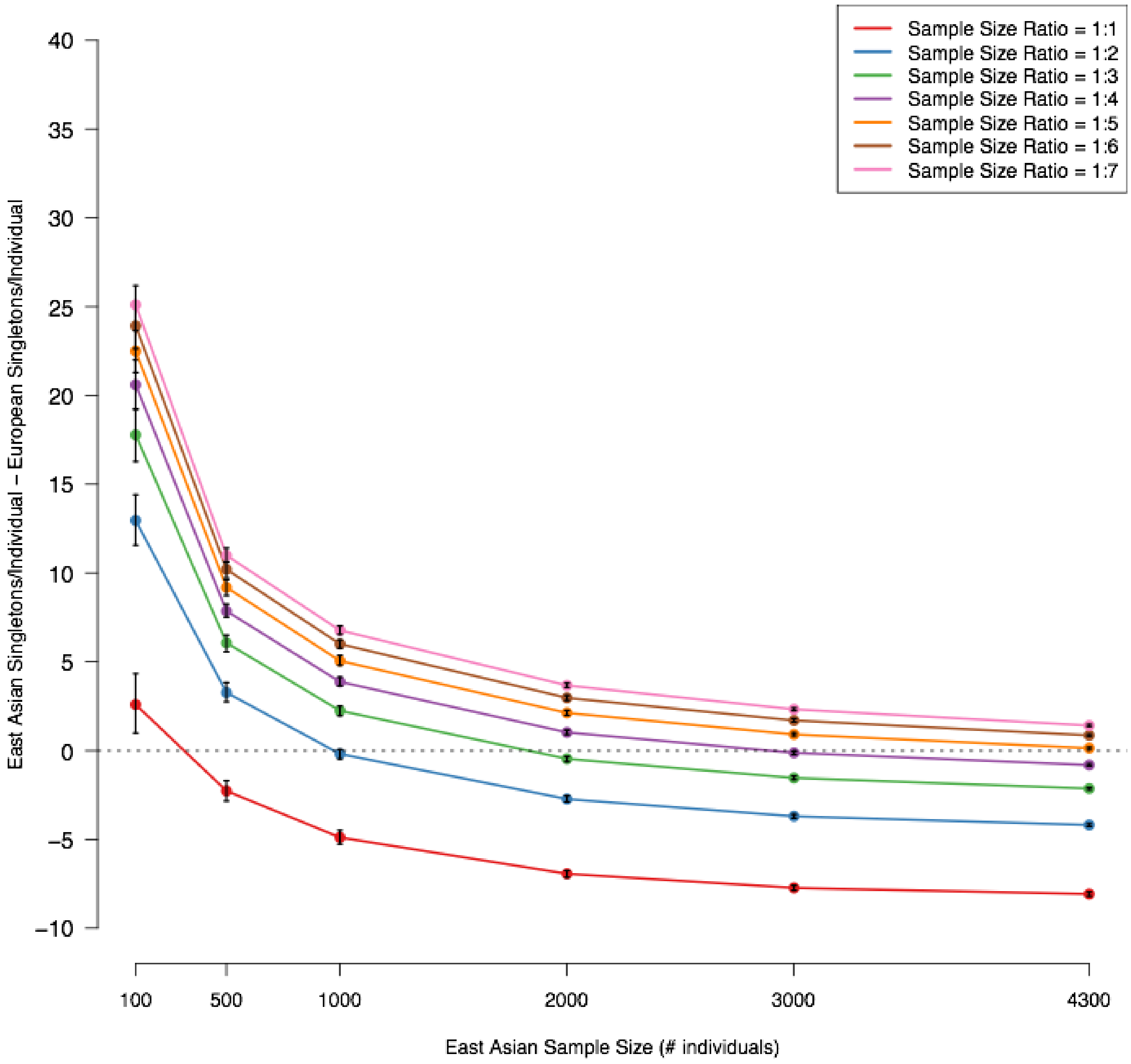

Supplement: Additional file 1: — Supplementary note and figures. The supplementary note describes the simulations we performed and the figures are analogous to the main text figures but represent data from simulations done with Latino, South Asian, and East Asian allele frequencies. (PDF 666 kb) [file 13059_2017_1172_MOESM1_ESM.pdf]
